# Supplementary material for: Identification of Potential Therapeutic Targets on the Level of DNA/mRNAs, Proteins and Metabolites: A Systematic Mapping Review of Scientific Texts’ Fragments from Open Targets
Source: Curr Issues Mol Biol. 2023 Apr 13;45(4):3406–18. doi: 10.3390/cimb45040223 (PMC10137072; doi:10.3390/cimb45040223)
Supplement: Supplementary file 1 [file cimb-45-00223-s001.zip › SupplementaryFile S1_questionary-nucleicAcids.html]

methodsGenomicsTranscriptomics


TOOL FOR CATEGORIZATION OF SHORT TEXT FRAGMENTS

*feed*Instruction


##### Text Fragment

To select text fragment enter its number:

Number

- *comment*Comments

  Comment


Does the text fragment allow to make a judgement on the category of experimental method?

NO

YES

Are highlited terms sufficient to judge on the category of experimental method?

NO

YES

Is DNA/RNA level study mentioned in the text fragment?

NO

YES


Next
Save


##### Categorization

- Load the text fragment.
- Read the text fragment.
- Name of the biological entity (protein, gene, metabolite) and terms related to the category of experimental method are highlited.
- Comment if necessary.
- Answer the questions.
- Go to the next fragment.
- Save the results.

Close
