# Supplementary material for: Identification of Potential Therapeutic Targets on the Level of DNA/mRNAs, Proteins and Metabolites: A Systematic Mapping Review of Scientific Texts’ Fragments from Open Targets
Source: Curr Issues Mol Biol. 2023 Apr 13;45(4):3406–18. doi: 10.3390/cimb45040223 (PMC10137072; doi:10.3390/cimb45040223)
Supplement: Supplementary file 1 [file cimb-45-00223-s001.zip › SupplementaryFile S3_questionary-metabolites.html]

methodsMetabolomics


TOOL FOR CATEGORIZATION OF SHORT TEXT FRAGMENTS

*feed*Instruction


##### Text Fragment

To select text fragment enter its number:

Number

- *comment*Comments

  Comment


Does the text fragment allow to make a judgement on the category of experimental method?

NO

YES

Are highlited terms sufficient to judge on the category of experimental method?

NO

YES

Is metabolite level study mentioned in the text fragment?

NO

YES

Next
Save


##### Categorization

- Load the text fragment.
- Read the text fragment.
- Name of the biological entity (protein, gene, metabolite) and terms related to the category of experimental method are highlited.
- Comment if necessary.
- Answer the questions.
- Go to the next fragment.
- Save the results.

Close
